# Wounds of Nerves

**Published:** 1838-01-03

**Authors:** Thomas Harris


### MEDICAL EXAMINER.

DEVOTED TO MEDICINE, SURGERY AND THE COLLATERAL SCIENCES.

EDITED by J. B. BIDDLE, M. D. and M. CLYMER, M. D.

No. 1. PHILADELPHIA, WEDNESDAY, JANUARY 3, 1838. Vol. I.

### WOUNDS OF NERVES.

*Notes of a Lecture delivered at the Philadelphia Medical Institute, by* Thomas Harris, *M. D., Surgeon of the Naval Asylum, and one of the Surgeons of the Pennsylvania Hospital; taken by one of the editors.**

It has been erroneously stated by authors, that the division of an important nerve will occasion a loss of sensibility and motion in the part to which it is distributed. Though this may be true when applied to the nerves of the extremeties, it is not the case so far as regards the face and head. The important discoveries of Sir Charles Bell have demonstrated that the facial nerve may be divided without producing any other effect than loss of muscular motion; the sensibility of the part being dependent on a branch of the fifth pair. If the latter nerve alone be divided, sensation is destroyed, whilst muscular action remains unimpaired.

This able physiologist has proved in a manner entirely satisfactory, that there exist, in the human system, besides the nerves distributed to organs of vision, hearing and taste, nerves of motion, sensation and respiration; and that these nerves have their origin in distinct tracts of nervous matter, either in the brain or spinal chord. He has further shown that "each filament, or tract of nervous matter has its peculiar endowment independently of the others which are bound up along with it, and that it continues to have the same endowment throughout its whole length. If we select a filament of a nerve, and if its office be to convey sensation, that power shall belong in all its course wherever it can be traced; and wherever, in the whole course of that filament, whether it be in the foot, leg, thigh, spine, or brain, it may be bruised, or pricked, or injured in any way, and sensation, not motion, will result; and the perception arising from the impression will be referred to that part of the skin where the remote extremity of the filament is distributed."

It is by attending to this view of the distributive character of nerves that we are able to explain the varied results ot the injuries to these chords.

As every part of the body is freely supplied by nerves, it is impossible to inflict a wound, however small, without dividing some filaments near their termination. It is not intended then to consider a division of the termination of nerves, which necessarily takes place in all lesions, but wounds of principal, or at least appreciable chords.

Wounds of nerves are commonly followed by immediate acute pain. If the nerve be only partially divided the pain is much more severe than when its continuity is entirely destroyed. A paralysis thus produced may be either temporary or permanent. If, by proper treatment, the nerve is healed by the first intention, its functions will in time be restored, and the paralysis removed. If, on the contrary, motion is permitted, which will interrupt the process of adhesion; inflammation, suppuration and ulceration will supervene, and a restorative process will not take place.

When the nerves of the senses are wounded they cannot be restored. Other nerves, which are simply divided, are capable of being re-united, and afterwards to perform their functions as well as before the wound was inflicted.

The experiments of Haighton, published in the British philosophical transactions of 1795, establish this point beyond controversy. These experiments have been repeated by Swan, of London, and Descot, of Paris, and with the same results. The phenomena connected with the re-union of a severed nerve, is the same ceteris paribus as those attending the restorative process in other tissues. The experiments to which I have referred, prove that when a nerve is divided, its extremities retract about half an inch; after twelve hours they elongate so as to diminish the separation more than one half. A deposition of a pulp like substance takes place, which fills up the interspace between the ends of the divided nerve. This, in time, by the recuperative powers of nature, assumes the appearance, and performs the office of the original structure.

When two or more inches of a nerve is destroyed, a deposit of new matter will be performed, but it will not be nervous tissue, nor will restoration of function in such instances follow.

In order that these injuries may be better understood, I will briefly notice several of them in succession.

### *Partial Division of Nerves.*

When a nerve has been thus injured, the patient complains of very acute pain, especially along the

* This lecture is published by permission of the accomplished lecturer, under whose revision it was prepared for publication.

course of the injured chord, and in the distant part to which it is distributed. When, for example, the sub-cutaneous nerve is wounded in the fold of the arm, in the operation of blood letting, severe pain is felt in the skin of the fore-arm and hand.

The painful effects of these wounds are sometimes so severe as to excite great constitutional irritation, and not unfrequently, tetanus. Frequently the pain is acute at the moment the wound is inflicted, but soon abates. Unless the part be kept perfectly at rest, the pain will return in three or four days, as violently as in the first instance. In other cases the suffering will continue with unabated severity, for weeks and months.

The treatment of incised wounds of nerves consists in promoting adhesion. With this view, rest should be strictly enjoined. In case of an injury of the sub-cutaneous nerve, at the fold of the arm, an angular splint should be applied to the arm, and secured there by a proper bandage. By such treatment the severe symptoms will not return after they have abated; but to secure this advantage, the splint should be immediately applied after the injury has been inflicted, and before the nerve becomes irritated by motion. When the nerve, from improper management, does become inflamed, the case always proves tedious, and accompanied with great suffering. To relieve it from this state, leeches, rest, anodyne embrocations, and time, will in most instances, accomplish a cure. When, however, there is no abatement of the symptoms, accompanied sometimes by convulsions, relief may be derived from completely dividing the nerve. This operation has been repeatedly performed with the effect of promptly affording relief to the patient. In two instances of partial division of the sub-cutaneous nerve, followed by very alarming symptoms, the patients were quickly relieved by my dividing the integuments and nerve by a free transverse incision. This operation has been repeatedly performed by others, and generally with the same success.

I saw a lady who had been bled in the foot, experience agonizing pain, followed by convulsive motions, who was promptly relieved by dividing the saphena nerve. Sabatier records a similar case, where he proposed this operation, but the patient not consenting, she was obliged to endure intense suffering for a period of six years.

Baron Larrey relates several cases which forcibly illustrates the importance of this practice. He states that a soldier was struck by a ball, which crossed his right arm, and wounded the biceps and coraco-brachialis muscles, and the radial and internal cutaneous nerve. The next day his local pains were very acute, convulsive motions of the hand and fore-arm, heat in the whole system, restless, and in continual agitation. The rapid progress of the symptoms determined Larrey to cut down to the bottom of the wound, where he found several nervous febrils, which he divided. This operation was very painful; but two hours afterwards the patient was very much relieved, and in two days all the symptoms disappeared.

Military Surgeons of all countries relate similar cases, which were treated successfully by the same means.

Punctured wounds of the fingers frequently cause very alarming symptoms, which are doubtless owing to a partial division of one of the nervous febrils which are so numerously distributed to these parts. These symptoms cannot result from a common wound, as an enlargement of it by the knife speedily removes them. The beneficial effects of such an operation can be only explained by supposing that it entirely divides the termination of a nerve which was partially wounded.

It is stated, by Wardrop, that a person pricked his forefinger by a gooseberry thorn, which caused the first phalanx to be so acutely painful that he could not endure it to be touched. After suffering severely for twelve months, the finger was amputated, by which all the unpleasant symptoms were removed.

Wounds of nerves, produced by bullets, never take on the restorative process. The nerve being contused, it sloughs like other tissues, and thus looses one or more inches of its continuity. The experiments of Swan, Descot, and others, have proved that when so much of the chord is destroyed re-union never takes place.

The following case, while it proves the truth of these remarks, affords a beautiful illustration of the views of Sir Charles Bell, in relation to the functions of the facial nerve.

In the action between the frigates Chesapeake and Shannon, Midshipman A—— received a pistol ball, which entered the anterior part of the ear, passed backward, and lodged in the base of the martoid process.

The muscles of the side of the face corresponding with the injury, lost entirely their motive powers, while the sensibility of the part became greatly exalted. The pain, indeed, continued so excessively violent that he was obliged to take, constantly, large doses of opium. His general health became impaired, and in six or eight years after the receipt of the injury he sunk under his accumulated sufferings.

After death I examined his wound, and found the ball lodged at the base of the mastoid process, and in contact with a branch of the fifth pair. In its course, it divided the facial nerve as it emerged from the stylo-mastoid foramen. The division of this nerve explains the cause of the paralysis, and the irritation of the nerve of sensation produced the violent pain which he had so long endured.

Pressure of a nerve will produce paralysis of the muscles to which it is distributed; it will also, sometimes, give rise to agonizing pain, and even tetanus. Haighton put a ligature around a nerve, and permitted it to remain until inflammation was established, ulceration ensued, and although re-union took place, the function of the nerve, in this instance, was not re-established.

I have known Dessault's apparatus for fractured clavicle, produce paralysis of the arm, by long continued pressure on the axillary plexus of nerves. This is by no means a solitary instance of the bad effects of this mode of treating such accidents.

